# Patterns of clinical presentation of adult coeliac disease in a rural setting

**DOI:** 10.1186/1475-2891-5-24

**Published:** 2006-09-14

**Authors:** Sián Jones, Charles D'Souza, Nadim Y Haboubi

**Affiliations:** 1 Dietetics, Royal Gwent Hospital. Newport, Gwent, South Wales, UK; 2 Specialist Registrar, Adult Medicine, Nevill Hall Hospital, Abergavenny, Gwent, South Wales, UK; 3 Consultant Physician, Adult Medicine, Nevill Hall Hospital, Abergavenny, Gwent, South Wales, UK

## Abstract

**Background:**

In recent years there has been increasing recognition that the pattern of presentation of coeliac disease may be changing. The classic sprue syndrome with diarrhoea and weight loss may be less common than the more subtle presentations of coeliac disease such as an isolated iron deficiency anaemia. As a result, the diagnosis of this treatable condition is often delayed or missed. Recent serologic screening tests allow non-invasive screening to identify most patients with the disease and can be applied in patients with even subtle symptoms indicative of coeliac disease. Both benign and malignant complications of coeliac disease can be avoided by early diagnosis and a strict compliance with a gluten free diet.

**Aim:**

The aim of this study is to evaluate the trends in clinical presentation of patients diagnosed with adult coeliac disease. In addition, we studied the biochemical and serological features and the prevalence of associated conditions in patients with adult coeliac disease.

**Methods:**

This is an observational, retrospective, cross-sectional review of the medical notes of 32 adult patients attending the specialist coeliac clinic in a district general hospital.

**Results:**

Anaemia was the most common mode of presentation accounting for 66% of patients. Less than half of the patients had any of the classical symptoms of coeliac disease and 25% had none of the classical symptoms at presentation. Anti-gliadin antibodies, anti-endomysial antibody and anti-tissue transglutaminase showed 75%, 68% and 90% sensitivity respectively. In combination, serology results were 100% sensitive as screening tests for adult coeliac disease. Fifty nine percent patients had either osteoporosis or osteopenia. There were no malignant complications observed during the follow up of our patients.

**Conclusion:**

Most adults with coeliac disease have a sub clinical form of the disease and iron deficiency anaemia may be its sole presenting symptom. Only a minority of adult coeliac disease patients present with classical mal-absorption symptoms of diarrhoea and weight loss. Patients with atypical form of disease often present initially to hospital specialists other than a gastro-enterologist. An awareness of the broad spectrum of presentations of adult coeliac disease, among doctors both in primary care and by the various hospital specialists in secondary care, is necessary to avoid delays in diagnosis. It is important to include serological screening tests for coeliac disease systematically in the evaluation of adult patients with unexplained iron deficiency anaemia or unexplained gastro-intestinal symptoms and in those who are considered to be at increased risk for coeliac disease.

## Background

Coeliac disease is an immunologically mediated enteropathy caused by a permanent intolerance to ingested gluten in genetically susceptible individuals [1-3]. Its prevalence rates in Caucasians in Europe, North and South America, Australia and the Middle East have been reported to be as high as 1 in 100 [4-6]. Typically, coeliac disease presents with symptoms of mal-absorption such as weight loss, diarrhoea, steatorrhoea, or abdominal distension. However, the symptoms of coeliac disease are diverse and it may present with a broad spectrum of clinical features such as isolated sub-clinical iron deficiency anaemia, osteoporosis, neurologic disease, non-specific abdominal symptoms, dermatitis herpetiformis or malignancies.

In recent years there has been increasing recognition that the mode of presentation of coeliac disease may be changing [7,8]. It often presents with symptoms not previously considered to be characteristic of the disease [3,9,10]. While most gastroenterologists appreciate the broader spectrum of the disease, and its increasing prevalence, it is still perceived by most general practitioners as a rare condition of childhood or infancy, presenting mainly with gastrointestinal symptoms suggestive of malabsorption [9]. There is concern that many patients with this disease are being overlooked due to failure of clinicians to consider it in the initial differential diagnosis when they present with non-classical symptoms [8]. These so-called "silent" coeliac disease patients lack diarrhoea and these non-diarrhoeal presentations now are seen more commonly than those with diarrhoea.

Early diagnosis of coeliac disease is important. In many patients with coeliac disease, the diagnosis is considered only when they present with avoidable complications such as a malignancy [11,12]. There is evidence that compliance with gluten free diet is protective against complications of coeliac disease, such as disorders of bone metabolism [13-15] and particularly non-Hodgkin's lymphoma [16], the most commonly associated malignant complication of coeliac disease [11].

## Methods

This is a retrospective study of patients attending specialist adult coeliac disease clinic in a district general hospital in rural South Wales. Case records of all patients with adult coeliac disease who attended three consecutive clinics were reviewed. This clinic was a specialist multidisciplinary adult celiac disease clinic run by a specialist physician and a specialist dietitian in a district general hospital in rural South Wales. The diagnosis of coeliac disease was based on compatible serologic tests, small bowel biopsy, and response to a gluten free diet. Adult coeliac disease was diagnosed if the clinical presentation was after 16 years of age. The case notes were reviewed regarding the following:

1. The clinical presentation including age at diagnosis, sex distribution, source and reason for referral; presenting symptoms, the time delay between onset of symptoms and the diagnosis, family history of coeliac disease or autoimmune disorders and the presence of any associated disorders including dermatitis herpetiformis.

2. The laboratory parameters at diagnosis including haematologic tests – full blood count, red cell folate, ferritin, serum B12; serological screen (Elisa technique) anti-gliadin antibodies (IgA), endomysial antibodies (IgA), anti-transglutaminase antibodies (IgA);

3. Small bowel biopsy result at diagnosis (Marsh classification);

4. Results of bone density scans – (dual energy x-ray absorptiometry: DEXA scan) – first scan following diagnosis.

If the relevant serological screening tests were not performed at the time of diagnosis, these cases were excluded from this study. Twelve (38%) anti-gliadin antibody values, 10 (31%) anti-endomysial antibody reports, and 4 (13%) anti-tTG results were not available; these patients were also excluded from the study. Four (13%) ferritin, 5 (16%) folate and 6 (19%) B12 test results were not obtainable, and these were excluded as well. In patients who were already on treatment for anaemia prior to the diagnosis of coeliac disease, haematological values before commencement of iron, B12 or folate replacement therapy were taken for analysis – as medication would have restored the subsequent values, thus creating a falsely low rate of anaemia at diagnosis, in these patients.

## Results

**Figure 1:**
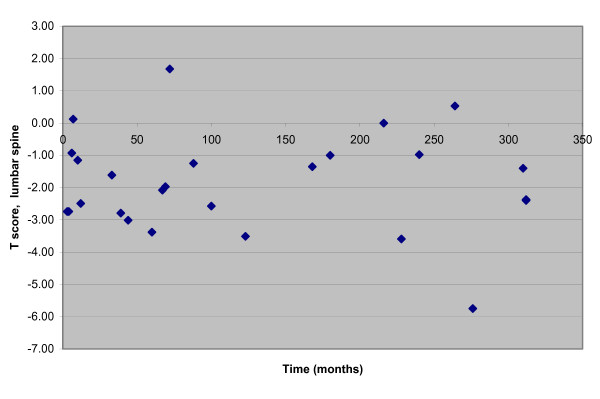
Time on GFD.

Case records of 32 consecutive adult coeliac disease patients attending three consecutive clinics fulfilled our criteria for inclusion in this study. The mean age at diagnosis was 53.2 years with a range of 23–86 years. There were two peaks in age at diagnosis, one in the 4^th ^and the second, in the 6^th ^decade (Figure 1). The ratio of male: female cases was 2:3. The duration of symptoms before diagnosis ranged from 1 week to 25 years, with a mean delay in diagnosis by 71 months. There was no correlation between age at diagnosis and time elapsed between onset of symptoms and diagnosis in this study (*r *= 0.18, *p *> 0.05), thus the possibility of age related diagnostic delay is effectively ruled out.

### Referral source

**Figure 2:**
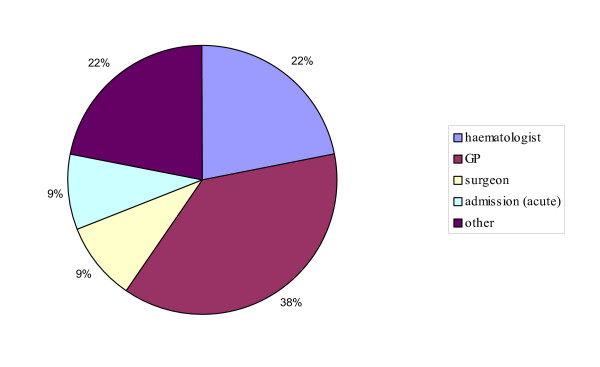
Source of referral.

Thirty eight percent of patients were referred from primary care by the general practitioners; most of the remaining patients were referred from secondary care: 9% were diagnosed during inpatient evaluation following an emergency admission to hospital with acute episodes of abdominal pain, diarrhoea or vomiting. The remaining patients were referred by hospital specialists other than gastroenterologists: 22% by a haematologist, 9% by a surgeon, 22% by general hospital physicians and care of the elderly specialists (chronic anaemia), or a gynaecologist (abdominal pain), dermatologist (skin rash) and rheumatologist (iron-deficiency anaemia) (figure 2).

### Presenting complaints

Anaemia was the most common mode of presentation: 68% patients had low iron stores (ferritin <20 ng/l) at diagnosis, 50% had a reduced haemoglobin, 35% had a low mean corpuscular volume (MCV) and 45% a low red blood cell count. 13% patients of our patients had anaemia with a high MCV at diagnosis; moreover, in some patients, a normal MCV might reflect a mixed nutritional anaemia since 57% our patients had either low folate or low B12 levels.

Less than 50% patients had at least one of the "classical" symptoms of coeliac disease; 25% (8 patients) had none of the "classical" symptoms at presentation and all these 8 subjects were found to have iron-deficiency anaemia. Only one patient (3%) had dermatitis herpetiformis although 25% cases suffered with skin conditions "diagnosed" as follicular rash, eczema, seborrheic dermatitis, lichen planus or pruritus of unknown aetiology.

### Co morbidities

Thyroid disorders were the most common disease associated with coeliac disease in this study. Amongst patients with thyroid disease, 57% had hypothyroidism and 29% were thyrotoxic while the remaining patient had thyroiditis. Eighty three percent of the cases with affective disorder had depression. Fifty seven percent of patients with associated arthritis had an inflammatory type of arthritis.

Fourteen percent of patients were noted to have selective IgA deficiency. The prevalence of pernicious anaemia was 9%, primary biliary cirrhosis 6%, and one patient had inflammatory bowel disease. Atopic disease was present in 6% of cases and one patient had sarcoidosis. No patients in this study had either type 1 diabetes mellitus or Sjögren's syndrome.

### Family history

Three patients had a positive family history with a diagnosis of coeliac disease in a first degree relative. In addition, 16% of our patients gave a history of other autoimmune diseases in the family.

### Serological screening tests

Seventy five percent of patients were found to have positive anti-gliadin antibodies (AGA) at diagnosis and 68% had positive endomysial antibodies (EMA) while 91% patients were positive for EMA, AGA or both (95% confidence interval 79.3–102.7%). 90% had positive anti-tTG (95% CI 71.4–108.5%). However, only eight patients had had all 3 antibody screening tests performed at the time of initial diagnosis, since these tests had not been widely available until much later.

### Histology

**Figure 3:**
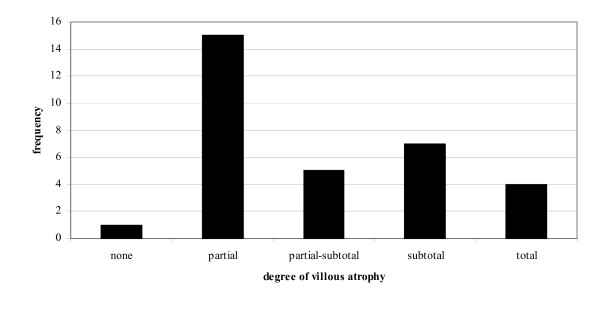
Findings at biopsy.

**Figure 4:**
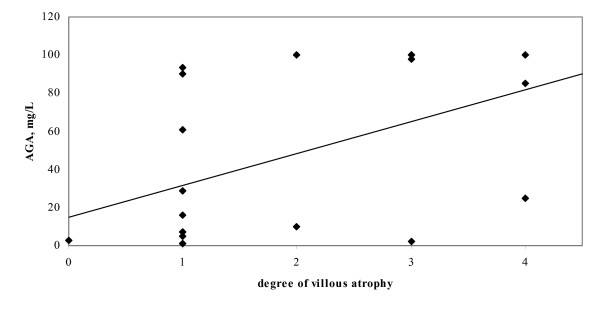
Association between degree of villious atrophy and AGA at diagnosis.

The most common finding (47%) was partial villous atrophy; 22% had subtotal villous atrophy and 16% partial-subtotal villous atrophy, while only 13% patients demonstrated total villous atrophy (figure 3). A statistically significant correlation was found between the degree of villous atrophy (no evidence of atrophy = 0, partial v.a. = 1, partial-subtotal v.a. = 2, subtotal v.a. = 3, total v.a. = 4) and AGA levels at diagnosis (*r *= 0.481, *p *= 0.013) (figure 4). The biopsy in one patient (3%) showed only an increase in the intraepithelial lymphocytes.

### Complications

Osteoporosis was the most common and also the only complication related to coeliac disease found among our 32 patients. Osteoporosis was present in 28%, with another 31% of patients were classified as osteopenic (osteoporosis = T score < -2.5, osteopenia = T score < -1.0). DEXA scans were carried out a mean of 124.7 months after onset of symptoms (and mean 43.4 months following diagnosis). There was no correlation between T score and the duration of symptoms before the DEXA scan (L-spine: *r *= -0.64, *p *= 0.757; hip: *r *= -0.108, *p *= 0.601), or between T score and the length of time elapsed following diagnosis before the DEXA scan (L-spine *r *= -0.320, *p *= 0.110; hip *r *= -0.0.279, *p *= 0.167).

There were a total four patients in our group, in whom a diagnosis of malignancy had been made on follow up: 2 with basal cell carcinoma, 1 each with breast cancer and melanoma. There were no cases of lymphoma, adenocarcinoma of the small intestine or oesophageal or pharyngeal carcinoma i.e. neoplasms thought to arise as a complication of coeliac disease.

## Discussion

Adult coeliac disease may present with classic clinical features including weight loss, diarrhoea, and malabsorption of nutrients. However, there are reports of an increasing trend towards silent or subclinical presentations i.e. presentation with subtle symptoms not clearly related to gastrointestinal system. Nonspecific symptoms and nutritional deficiencies are especially common in older patients and as a result, the diagnosis of this treatable condition is often delayed or missed. Without active serologic screening, most cases of coeliac disease probably remain undiagnosed. The mean age at diagnosis in this study was 53.2 years with peaks in the 4^th ^and 6^th ^decades. Similar age distribution has been reported in other studies [8,10]. Coeliac disease is more common in women than men [17,18] and the cases presented in this study concur with this, with a male: female ratio of 2:3. It is known that women actively seek health advice more readily than men [7,19], and the high relative incidence in women may thus be a result of this behavioural pattern. It is also possible that the higher incidence in women could be due to the increased nutritional demands of women during the reproductive years [7]. This is supported by the finding that in children the ratio of boys: girls with coeliac disease approaches 1: 1 [7,20].

Only thirty eight percent of cases in this study were referred directly from primary care by their general practitioners. Twenty two percent of patients were referred directly by a haematologist, reflecting the high incidence of anaemia among the group.

Delays in diagnosis of coeliac disease and initiation of a gluten-free diet may predispose patients to developing complications [13-15]. It has been shown that adherence to a strict gluten-free diet is protective against malignancy in coeliac disease [16]. A cohort study of 210 patients with coeliac disease who followed a gluten-free diet for a mean of 18.5 years, found that the risk of developing cancer is not significantly increased in those who followed a gluten free diet for five years or more when compared with the normal population [16]. This study found however, that those taking a reduced gluten or normal diet had an increased risk of developing cancer, especially lymphomas and cancers of the mouth, pharynx and oesophagus.

Disorders of calcium and bone metabolism were the most prevalent complications seen in coeliac disease in our study. Thirty one percent were osteopenic while 28% had osteoporosis and one patient had secondary hyperparathyroidism. In patients with coeliac disease, delay in diagnosis and hence delayed treatment leads to an increased risk of development of osteopenia or osteoporosis [13]. In addition, there is evidence to show that compliance with a gluten-free diet increases the bone mineral density of osteopenic coeliac patients [14,15].

The most common presenting complaint in this study was iron-deficiency anaemia. Non-specific abdominal pain or discomfort, often longstanding was also a common presenting symptom. Some of our patients, who had non-specific abdominal pain as the primary symptom, had undergone prolonged investigations elsewhere, before the diagnosis of coeliac disease was considered. Six of these 13 patients with non-specific abdominal pain had had a chronic undiagnosed anaemia for years before developing gastrointestinal symptoms which eventually led to their diagnosis.

Zipser et al. [10], in their study conducted in the USA reported fatigue to be one of the most common presenting symptom, with 82% patients complaining of this. However, fatigue was not seen as a significant symptom in our study and it was reported in only 31% of case notes. A comparison between the findings of this study and those by Zipser et al. are shown in table 1.

**Table 1:** Comparison between the presenting complaints of patients in this study compared to those in the study of Zipser et al.^10^

**Complaint**	**Current study (%)**	*Zipser et al (%)*
Anaemia	66	63
Abdominal pain or discomfort	41	77
Diarrhoea	38	52
Fatigue	31	82
Nausea and/or vomiting	28	46
Weight loss	28	55
Depression	19	46
Generalised aches and pains	19	42

Although 57% patients had either low folate or low B12 levels at the time of diagnosis, a raised MCV was seen only in a minority of patients (13%). However, 91% of those patients with a normal MCV despite low folate or B12 levels also had low ferritin levels, reflecting their mixed deficiency anaemia, thus explaining their normocytic blood film.

Dermatitis herpetiformis was seen in only one patient in this study. However, since the diagnosis of dermatitis herpetiformis is made by skin biopsy showing pathognomonic granular IgA deposits in the papillary dermis in apparently unaffected skin, with increased numbers of activated T cells [21], an accurate diagnosis is unlikely to be made unless it was specifically suspected by the physician. Thus the follicular rashes, seborrhoeic dermatitis, lichen planus and pruritis recorded in the case-notes in some of our patients may actually represent undiagnosed cases of dermatitis herpetiformis.

Family history was considered as positive if any first degree relative of our patients also had a diagnosis of coeliac disease and three cases met these criteria. In family screening studies, around 10% of first degree relatives of coeliac disease patients are diagnosed with coeliac disease[8]. However, since we did not perform routine serological screening tests on the first degree relatives, we would have missed undiagnosed, sub-clinical or silent celiac disease in the family.

Sixteen percent of our patients had a first degree relative with an autoimmune disorder. The prevalence of autoimmune disorders among relatives of coeliac disease patients has been shown to be higher than among the general population. It was also shown that those relatives with an autoimmune disease were more likely to have silent, rather than symptomatic, coeliac disease [22].

The most common associated disorder seen in our patients presented here was thyroid disease (22% patients affected). There is a well-recognised association between these two diseases [3,11,23,24]. One explanation for the association between coeliac disease and thyroid disorders is the coexistence of HLA molecules in both diseases [23] and HLA DR3 [23,24], HLA B8 [23] and HLA DQ8 [24] have been implicated. An alternative hypothesis is that the autoimmune reaction which occurs in the presence of gluten in coeliac disease patients promotes the development of autoimmune disorders [25]. Furthermore, it has been shown that these auto-antibodies disappear on withdrawal of gluten from the diet [26]. Many of the coeliac disease associated disorders are autoimmune in nature and 34% of patients in our study were found to have a coexisting autoimmune disorder. These results are similar to those reported by Collin et al. [27], where 28% coeliac disease patients had an associated disorder considered to be of autoimmune origin.

Arthritis and affective disorders were jointly the second most common associated disorder found among patients with coeliac disease in this study. Although, rheumatoid arthritis is frequently quoted as being coeliac-associated [17,28,29], there is little evidence in the literature to support this association [30,31]. However, there is some evidence to support the concept of an associated enteropathic arthritis in patients with coeliac disease as has been suggested by Lubrano et al. [29]

Nineteen percent of patients in this study had been diagnosed with an affective disorder. Fera et al. [32] suggest that affective disorders in coeliac disease are reactive, resulting from the impact of initial diagnosis and subsequent adjustment to a gluten-free diet. They recommend preventative supportive psychologic interventions in patients with a new diagnosis of coeliac disease.

The association between coeliac disease and IgA deficiency is widely recognised [27,33,34]. and this immunological disorder is common among coeliac disease patients. The reason for this association is unclear and it is not certain whether this is due to a common genetic factor or whether one condition predisposes to the other. The HLA alleles B8, DR3, DR7 and DQ2 are thought to be likely candidates for a genetic cause as they are prevalent in both conditions [27,33]. The primary concern regarding this association is the potential for a false negative serological tests for coeliac disease and hence missed diagnosis of coeliac disease as serological tests become the principal form of screening. Thus Gillett et al. [34] have argued that small bowel biopsy confirmation of diagnosis becomes essential especially in patients with selective IgA deficiency.

Although the associations between type 1 diabetes mellitus and Sjögren's syndrome with coeliac disease has been well documented [3,8,11,20,23] neither of these disorders were found among patients in this study. This is most likely attributable to our small sample size.

Pernicious anaemia and primary biliary cirrhosis are among the less frequently reported disorders associated with coeliac disease; although, they were both relatively prevalent among the patients studied here (9% and 6% respectively). Three cases of pernicious anaemia were found in our study; the association between pernicious anaemia and coeliac disease has been somewhat underreported in the literature [35].

Elevated transaminase levels is frequently found at diagnosis in patients of coeliac disease and liver cell damage, characterised by inflammation and steatosis, has been shown to resolve on a gluten-free diet [36,37]. The relationship between inflammatory bowel disease and coeliac disease is well recognised [38]. Inflammatory bowel disease is more prevalent among first degree relatives of coeliac disease patients than the normal population [39]. However, only one of our 32 patients studied here had proven ulcerative colitis, and Crohn's disease was not seen in any.

There is a possible association between coeliac disease and sarcoidosis [23,40]. One patient in this study had sarcoidosis which, given the rarity of this disease in this age group and in this population, may support the suggestion of association between these two disorders.

Small intestinal biopsy was diagnostic in 97% patients and was equivocal in only one patient who had an increase in intraepithelial lymphocyte reported in his duodenal biopsy. In this isolated case anti-tTG was significantly raised at 120.3 U/ml (reference range 0–10). This patient had classical symptoms of abdominal discomfort and diarrhoea noted to occur following ingestion of wheat. He improved on a gluten free diet; his symptoms disappeared; and his serology normalised. In patients with positive small intestinal biopsies, it is to be noted that most patients did not a have total villous atrophy: the majority had only a partial villous atrophy. There was a statistically significant association between the histological grade and anti-gliadin antibody levels at diagnosis. This would add support to the argument of some who believe that serologic tests should now be the preferred test, to be used in the initial screening of patients thought to be at risk for coeliac disease [41].

Serological screening tests have improved case detection of coeliac disease in patients with atypical presentation. This study showed the sensitivity of EMA to be 68% and that of AGA to be 75%. In combination these two antibodies gave 91% sensitivity (95% CI 79.3–102.7%). The sensitivity of tissue anti-tTG was 90% (95% CI 71.4–108.5%). This demonstrates the tests to be 100% sensitive in combination, even in this study with a small sample size. Other studies have shown EMA to be 68–100% specific [42]; and when used in conjunction with AGA, a 98% sensitivity has been reported [8]. Thus the results obtained here do not differ significantly from those reported in previous studies [43].

Serological tests may be false-negative in cases of IgA deficiency and hypogammaglobulinaemia [44]. Although there were no IgA-deficient patients in the study, one patient had hypogammaglobulinaemia. This patient however was not tested for anti-tTG at the time of diagnosis but did have positive EMA and AGA.

With the introduction of serologic tests for coeliac disease, disease variants have been recognised which present with atypical features such as fatigue, asymptomatic iron deficiency, decreased bone density and dyspepsia without the classic malabsorption syndrome. Other presentations could include isolated hypoalbuminaemia, elevated aminotransferase levels (transaminitis), microscopic colitis, symptoms of irritable bowel syndrome, recurrent aphthous stomatitis, infertility, neurologic symptoms such as peripheral neuropathy, ataxia and epilepsy with posterior cerebral calcification. The findings of this study suggest that patients presenting to their general practitioner with unexplained iron deficiency anaemia, unexplained fatigue or generalised abdominal pain should undergo serological testing for coeliac disease.

## Conclusion

Most adults with coeliac disease have a sub-clinical form of the disease and iron deficiency anaemia may be its sole presenting symptom. Only a minority of adult coeliac disease patients present with classical mal-absorption symptoms of diarrhoea and weight loss. Patients with atypical form of disease often present initially to hospital specialists other than a gastro-enterologist. It is important to include serological screening tests for coeliac disease systematically in the evaluation of adult patients with unexplained iron deficiency anaemia or unexplained gastro-intestinal symptoms and in those who are considered to be at increased risk for coeliac disease.

An awareness of the broad spectrum of presentation of patients with coeliac disease among doctors both in primary care and by the various hospital specialists in secondary care is necessary to avoid delays in diagnosis of patients with adult coeliac disease. Adherence to a strict gluten free diet is known to markedly reduce the risk of coeliac disease related complications.

## Competing interests

The author(s) declare that they have no competing interests.

## Authors' contributions

SJ, CDS, and NYH contributed equally to this study. All authors read and approved the final manuscript.
